# Spatial and Spatio-Temporal Models for Modeling Epidemiological Data with Excess Zeros

**DOI:** 10.3390/ijerph120910536

**Published:** 2015-08-28

**Authors:** Ali Arab

**Affiliations:** Department of Mathematics and Statistics, Georgetown University, 37th and O streets, Washington, DC 20057, USA; E-Mail: ali.arab@georgetown.edu; Tel.: +1-202-687-1878; Fax: +1-202-687-2662

**Keywords:** spatio-temporal models, spatial models, hierarchical modeling, Bayesian analysis, zero-inflated models, hurdle models, Integrated Nested Laplace Approximation (INLA)

## Abstract

Epidemiological data often include excess zeros. This is particularly the case for data on rare conditions, diseases that are not common in specific areas or specific time periods, and conditions and diseases that are hard to detect or on the rise. In this paper, we provide a review of methods for modeling data with excess zeros with focus on count data, namely hurdle and zero-inflated models, and discuss extensions of these models to data with spatial and spatio-temporal dependence structures. We consider a Bayesian hierarchical framework to implement spatial and spatio-temporal models for data with excess zeros. We further review current implementation methods and computational tools. Finally, we provide a case study on five-year counts of confirmed cases of Lyme disease in Illinois at the county level.

## 1. Introduction

It is common to encounter the problem of having a large proportion of zero values in many physical processes, including those in epidemiological, ecological and environmental studies. In particular, epidemiological studies often include counts of cases of a certain condition or disease, incidence rates, and sometimes presence/absence of epidemiological phenomena (e.g., see [1]). The zero valued data should not be ignored and dropped from the analysis as they often provide important information regarding the process. Having a large proportion of zeros could indicate important characteristics of the disease or condition under study including its prevalence in the population, detectability (or capture rate), and population awareness regarding the disease. Thus, specific probability models that are capable of handling excess zeros should be considered as standard models such as Poisson, binomial, and negative binomial often poorly fit data with excess zeros due to inability to generate similar levels of zero and non-zero values. To this end, there has been an increase in popularity for classes of “zero-inflated” and “hurdle” distributions which can properly account for a large proportion of zero values while modeling non-zero data [2,3,4]. Specifically, discrete zero-inflated and hurdle models have become popular tools for handling count data with excess zeros.

Recently, there has been increasing interest in modeling epidemiological data with excess zeros, particularly for data on rare diseases, and diseases and conditions with heterogeneous spatial impact (e.g., Lyme disease). Moreover, often it is critical to understand and account for spatial and spatio-temporal dependence structures in the data whether the goal is to conduct inference or prediction. In this paper, we present a review of existing methodology for modeling spatial and spatio-temporal epidemiological data with excess zeros. We also provide a case study of confirmed cases of Lyme disease in the state of Illinois and discuss appropriate modeling strategies.

The remainder of the paper is structured as follows: Section 2 provides a review of common models for data with excess zeros, namely, hurdle models and zero-inflated models, as well as spatial and spatio-temporal extensions for these models. Section 3 discusses a case study on 2007–2011 confirmed cases of Lyme disease for the state of Illinois. Section 4 provides results and discussion. Finally, conclusions are provided in Section 5.

## 2. Models for Data with Excess Zeros

In this section, we review common modeling approaches for data with excess zeros. In particular, we review hurdle and zero-inflated models and spatial and spatio-temporal extensions of these models. Hurdle and zero-inflated models can be viewed as mixture models. Although the two approaches are similar, there are subtle differences between them as we discuss below.

### 2.1. Hurdle Models

A hurdle model [5,6] is a two-component mixture model of a distribution that generates non-zero values, and a point mass at zero. It should be noted that hurdle models have a general definition than what we use here for excess zeros and the “hurdle” may be any value and not necessarily zero. However, in practice, most often hurdle models are used with the hurdle value at zero [4]. The two components of a hurdle model include a binary component that generates zeros and ones (“zeros” correspond to zero values in data and “ones” correspond to non-zero values in data), and a second component which generates non-zero values (often positive values from a zero-truncated distribution).

Under this model, it is assumed to be a two-stage process that generates the zero and non-zero data. It is assumed that all the zero-valued data are generated through a single process (all zeros are assumed to be “structural” zeros, e.g., condition is absent and thus a zero is observed). For example, a Poisson hurdle model for the set of *n* independent and identically distributed (*iid*) observations $Y_{i}$’s (for i=1,…,n) can be described as the mixture of a point mass at zero with probability *p* and a zero-truncated Poisson distribution with probability (1−p):

(1) $$\begin{array}{l} P\left(Y_{i}=0\right)=p,\;0\leq p\leq 1\\ P\left(Y_{i}=k\right)=\left(1-p\right)\frac{\mu^{k}e^{-\mu }}{k!\left(1-e^{-\mu }\right)},\;k=1,2,\ldots ,+\infty ,\mu >0 \end{array}$$

where, $Y_{i}$ is the *i*-th response and μ is the mean of the untruncated Poisson distribution. This definition may be extended so that a log-linear regression model be considered for observation-specific means, $\mu_{i}$’s based on predictor variable(s) information. Similarly, a logistic regression may be considered for observation-specific probabilities, $p_{i}$’s.

### 2.2. Zero-Inflated Models

A zero-inflated model [3,7] is a mixture of a distribution (e.g., the Poisson distribution) and a point mass at zero. Under this model, it is assumed that two types of zeros may occur through two processes, either by definition (*i.e.*, a process that generates only zeros—condition is absent and thus, a zero is recorded), or by chance (*i.e.*, a process that generates both zeros and non-zeros—condition is present but in some cases may not be detected and thus, a zero is recorded by “mistake”). For example, the zero-inflated Poisson (ZIP) can be described as the mixture of a point mass at zero with probability *p* and a Poisson distribution with probability (1−p):

(2) $$\begin{array}{l} P\left(Y_{i}=0\right)=p,\;0\leq p\leq 1\\ P\left(Y_{i}=k\right)=\left(1-p\right)\frac{\mu^{k}e^{-\mu }}{k!},\;k=0,1,2,\ldots ,+\infty ,\mu >0 \end{array}$$

Similar to the hurdle model, for the *i*-th response, $Y_{i}$, using predictor variables, a log-linear regression model may be considered for observation-specific means, $\mu_{i}$’s, and also, a logistic regression may be considered for observation-specific probabilities, $p_{i}$’s.

### 2.3. Model Choice between a Hurdle Model and a Zero-Inflated Model

The choice between a zero-inflated model and a hurdle model is often dependent on the nature of the problem. Although these two models are similar in many aspects, conceptually there is a subtle difference between the two models and depending on the application and the data collection procedures, one may be more appropriate than the other [4]. Despite differences between the modeling frameworks (the hurdle model includes a mass at zero and a truncated distribution whereas the zero-inflated model is based on a mass at zero and a “regular” distribution), the inferential results are often very similar. Hurdle models are more general in the sense that they can handle both cases where there are fewer or more zeros than assumed by a “regular” distribution. Also, the “hurdle” does not necessarily have to be set at “0”. Zero-inflated models, although less general than hurdle models, are sometimes preferred due to the assumption that two different types of zeros (“structural” or true zeros, *vs.* “sampling” zeros) may exist in the data. It is recommended that hurdle models are more appropriate for cases where a real separation of mechanisms producing the zeros and the positive counts is justified [4]. Otherwise, zero-inflated models are more appropriate (due to lack of information or knowledge regarding the non-existence of overlap between the two potential sources of zeros). In practice, often due to lack of clear evidence regarding the nature of zeros, model selection procedures are implemented in order to arrive at the best model choice.

From a technical standpoint, the two methodologies are substantially different since hurdle models are two-stage models (the algorithms for fitting the model for the binary component and the non-zero data component are implemented separately) while zero-inflated models fall under the class of finite mixture models (the parameters for zero and non-zero parts of the models are estimated simultaneously). Consequently, the interpretation of parameter estimation results may be difficult across these two models due to differences in model structures.

### 2.4. Spatial and Spatio-Temporal Models with Excess Zeros

Over the past few decades and with the advent of computational methods and statistical methodology, and availability of spatially-referenced data and software tools, spatial and spatio-temporal modeling have increased in popularity in epidemiological research [1,8]. Particularly for modeling infectious and rare diseases, as well as diseases and conditions with direct link to environmental factors (e.g., temperature, humidity, precipitation, *etc.*), it is critical to consider spatial or spatio-temporal variabilities in order to reliably conduct inference or prediction about the process under study. In many cases, these types of data include excess zeros. Recently, there has been an increase in studies which consider spatial and spatio-temporal structure, and excess zeros in the epidemiology. This is mostly due to the recent popularity of methodology and availability of software tools. In this section, we describe straightforward approaches to implementing spatial and spatio-temporal hurdle/zero-inflated models.

Here, we advocate a Bayesian hierarchical modeling approach [9,10] where a complex problem is broken down into three stages: data model(s), process model(s), and parameter models. This approach allows us to conveniently account for data sampling variability, parameter uncertainty, and potential dependence structures such as spatial and temporal structures. Bayesian inference is natural for the hierarchical modeling framework, however, non-Bayesian methods may be used too [10]. Using a Bayesian approach, we can obtain the joint posterior distribution for process and parameters given data. This is often facilitated using Markov Chain Monte Carlo (MCMC) or other similar numerical procedures to draw from the posterior distribution (due to lack of an analytical solution). However, methods to approximate the posterior distributions are also available. Mainly, the integrated nested Laplace approximation (INLA) has gained popularity as an approximation tool for fitting Bayesian models. Further discussion on software implementation tools will be provided in Section 2.5.

Common choices to account for spatial structure include distance-based exponential or Matérn covariance functions for geostatistical data, and conditionally autoregressive (CAR) models for areal data [11,12]. However, other choices may be considered and easily implemented through the hierarchical modeling framework [11]. In spatio-temporal settings, it is often assumed that the covariance is separable in space and time, and thus, the temporal structure may be modeled using an autoregressive process. Cressie and Wikle [11] provide a detailed review of these methods.

There are many examples of spatial and spatio-temporal models for data with excess zeros in the literature (e.g., see [13,14]). Recently, these methods have gained popularity in epidemiology and public health studies. For example, Neelon *et al.* [15] use a spatial hurdle model to explore geographic variation in emergency department visits. Oleson and Wikle [16] use a spatio-temporal hurdle model based on a Gaussian latent process to predict infectious disease outbreak risk via migratory waterfowl vectors. Amek *et al.* [17] implement a spatio-temporal zero-inflated binomial model for malaria sporozoite rates. Musenge *et al.* [18] uses the INLA approach to implement Bayesian spatial and spatio-temporal zero-inflated models.

Here, we define a general hierarchical model for count observations $Y_{i}$’s (for i=1,…,n) and predictor variables $X_{i},\ldots ,X_{p}$. Note that the observations are not *iid* due to spatial or spatio-temporal structure. In the first stage, the data model is defined, and the advantage of the hierarchical modeling framework is that it allows us to consider *conditional independence* of observations at this level (*i.e.*, the dependence structure will be defined at a different level of the hierarchical model). The general hierarchical model can be described as follows:

(1) Data Model

(3) $$Y_{i}∼f\left(y_{i}|\theta_{i},p\right),\;i=1,\ldots ,n$$

where $f\left(y_{i}|\theta_{i},p\right)$ is a hurdle or zero-inflated distribution with parameters $\theta_{i}$’s and mixture probability *p*.

(2) Process Model

(4) $$g\left(\theta_{i}\right)=\beta_{0}+\beta_{1}X_{1i}+\ldots +\beta_{p}X_{pi}+\gamma_{i},\;i=1,\ldots n$$

where g(.) is a function defined according to the conditions on $\theta_{i}$’s. This is called the “link function” for *generalized linear models* (e.g., *log* function for the Poisson data model). Similarly, a regression model (often a logistic regression) can be considered for the mixture probabilities. Parameters $\beta_{i}$’s are the regression coefficients for predictor variables $X_{1},\ldots ,X_{p}$. Parameters $\gamma_{i}$’s are spatially correlated error terms. Let $\gamma =\left(\gamma_{1},\ldots ,\gamma_{n}\right)$ be the vector of spatially correlated errors such that:

(5) *γ* ~ *N*(0,Σ)

where Σ is the covariance matrix which describes the spatial dependence of the data. The covariance matrix Σ should be defined based on appropriate choices for geostatistical or areal data. For example, for geostatistical data, the covariance matrix can be defined as:

(6) $$\text{Σ}=\sigma^{2}R\left(\tau \right)$$

where the spatial correlation is defined based on an exponential covariogram model which is a common choice [11,12]. Thus:

(7) R(τ)=exp(−τd)

where the spatial correlation is symmetric and is based on the Euclidean distance between data points (*d*) and a spatial range parameter, τ. Many other options for the spatial correlation are available [11,19].

Similarly, for modeling spatio-temporal structure in the data, temporally correlated error terms may be considered. A common approach to model temporal correlation is through autoregressive models (e.g., see [18]). However, more complex methods may be used such as those for dynamical spatio-temporal models [11].

(3) Parameter Models

In the Bayesian setting, parameter models are the prior distributions for the unknown parameters (e.g., $\beta_{i}$’s, τ, and $\sigma^{2}$). Appropriate prior densities should be defined based on the characteristics of the parameters. If *a priori* knowledge is available, the prior distributions may reflect that, otherwise, it is recommended to assign relatively non-informative prior distributions to parameters.

For high dimensional spatial and spatio-temporal models problems, dimension reduction techniques may be considered including low rank methods and “predictive process” (see [11] for a detailed review). Finally, these methods may be extended to multivariate cases (e.g., see [20] for an example of bivariate ZIP model).

### 2.5. Software Tools and Implementation

Spatial and spatio-temporal hierarchical hurdle/zero-inflated models can be fitted to data using high-level programming languages (such as R and MATLAB (Mathworks, Natick, MA, USA)) or low-level languages (such as C, C++, FORTRAN). More common alternatives include implementations using off-the-shelf MCMC and Gibbs sampling tools such as BUGS. The freely-distributed Bayesian computation software WinBUGS (http://www.mrc-bsu.cam.ac.uk/bugs/) or its open source version, OpenBUGS (http://www.openbugs.net/w/FrontPage), and its spatial package GeoBUGS can be used to carry out Bayesian computations [12]. Similarly, JAGS (http://mcmc-jags.sourceforge.net/) can be used. JAGS is based on BUGS but unlike WinBUGS/OpenBUGS (which are limited to Windows), it is available on many platforms. A more recent computational tool that is becoming popular is the integrated nested Laplace approximation (INLA; http://www.r-inla.org/; [21]). The INLA approach is a numerically implemented analytical solution for approximating posterior marginals in hierarchical models with latent Gaussian processes. In this paper, we perform computations based on R-INLA package (an implementation of INLA method in R).

INLA is a very useful tool for fitting spatial and spatio-temporal models. INLA allows relatively simple implementation of both hurdle and zero-inflated models (Poisson, binomial, and negative binomial versions) including spatial and spatio-temporal versions of these models [22]. However, INLA does not allow fitting a regression model for the zero-inflation probability of the zero-inflated models. This can be done (with some extra coding) for the hurdle model. INLA allows spatial modeling for both geostatistical and areal data. Also, see Quiroz *et al.* [23] for a spatial hurdle model with continuous skewed positive data with excess zeros (in this case, a gamma hurdle model is considered, but other choices including log-normal and log-logistic are possible too). INLA also, allows a simple model selection procedure using the Deviance Information Criterion (DIC; [24]). In the next section, we present an example case study with spatial hurdle/zero-inflated models fitted using R-INLA.

## 3. Case Study: Lyme disease in Illinois

Lyme disease is a bacterial disease caused by the spirochaete *Borrelia burgdorferi* and transmitted by infected ticks [25]. It was first recognized in the United States in 1975 after a mysterious outbreak of arthritis near Old Lyme, Connecticut. Since then, reports of Lyme disease have increased dramatically, and the disease has become an important public health problem. Lyme disease is prevalent in the Eastern United States [26].

Here, we consider modeling Lyme disease counts in Illinois. We have data on five-year total of confirmed cases of Lyme disease aggregated at the county level for the state of Illinois. The counts are aggregated over years 2007 to 2011. Although, data are aggregated over time and space, there are still many zero values given that Lyme disease is not common in Illinois. Data are obtained from the Center for Disease Control (CDC) and only include non-zero values given that the confirmed cases are reported. We assume the unreported cases to be zeros.

**Figure 1:**
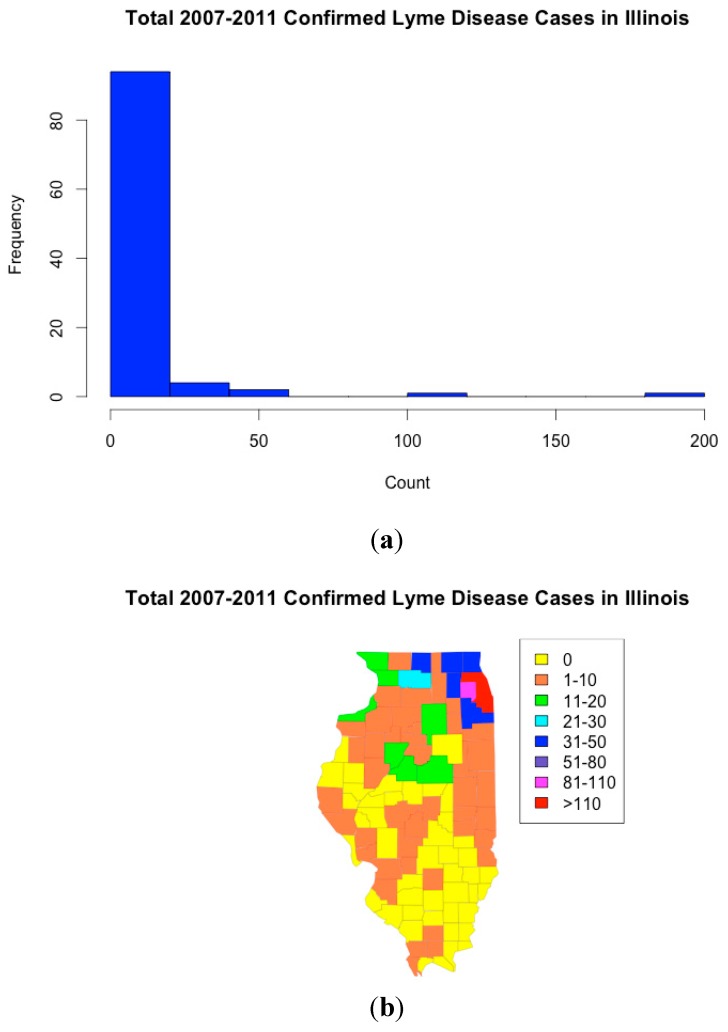
Histogram (**a**) and map (**b**) of the total number of confirmed cases of Lyme disease in Illinois by county for the 5–year interval 2007–2011.

There are a total of 102 observations and each observation represents the number of confirmed cases in each county of Illinois aggregated over years 2007 to 2011. Figure 1 shows a histogram as well as a map of the data. There are a few counties with large values, namely Cook and DuPage counties with 186 and 102 confirmed cases over the 5-year period have the highest values in the set, respectively. Cook County is the most populous county in Illinois (Chicago is the county seat). DuPage County is a neighboring county to Cook County (and a suburb of Chicago). Similarly, most of the counties with relatively large confirmed cases are clustered around Cook County. In general, other than a small cluster of counties with relatively large confirmed cases (Peoria County, 14 cases; McLean County, 13 cases; and Tazewell County, 11 cases), all the counties with large number of cases are in the northern part of the state. Most of the southern counties have zero values or low non-zero values (no southern county has more than three cases). Although not common across the state, Lyme disease is becoming common in the Northern parts of Illinois. For example, according to CDC [27] the number of confirmed cases in Illinois has more than doubled between years 2004 and 2011 (from 87 to 194) with a consistent pattern of high activity in the Northern part of the state. We also use elevation (in ft.), which is measured based on the maximum elevation within the county, and population per square mile for each county (calculated based on the 2010 Census) as potential predictor variables for both zero and non-zero components of the models. It should be noted that the zero and non-zero components do not have to include the same set of predictor variables. Additionally, we use the geographical coordinates of the county seats to account for spatial structure of the data.

We consider both the Poisson hurdle and the zero-inflated Poisson models. However, given that Lyme disease is not prevalent in Illinois, it is not likely that the zeros are generated based on two processes (e.g., true zero counts *versus* “false” zeros due to detectability issues). Thus, a hurdle model seems to be appropriate. We will fit a spatial hurdle model with log-linear regression for the Poisson intensity, and a spatial hurdle model with both a log-linear for the Poisson intensity and a logistic regression for the zero-inflation probability (see Supplementary File 1 for more details about the models). We also fit a spatial zero-inflated Poisson model with log-linear regression for the Poisson intensity. As part of our model selection, we also considered negative binomial hurdle and zero-inflated models, especially since a few recent studies have considered negative binomial models for Lyme disease data (see [28] and references therein).

**Figure 2:**
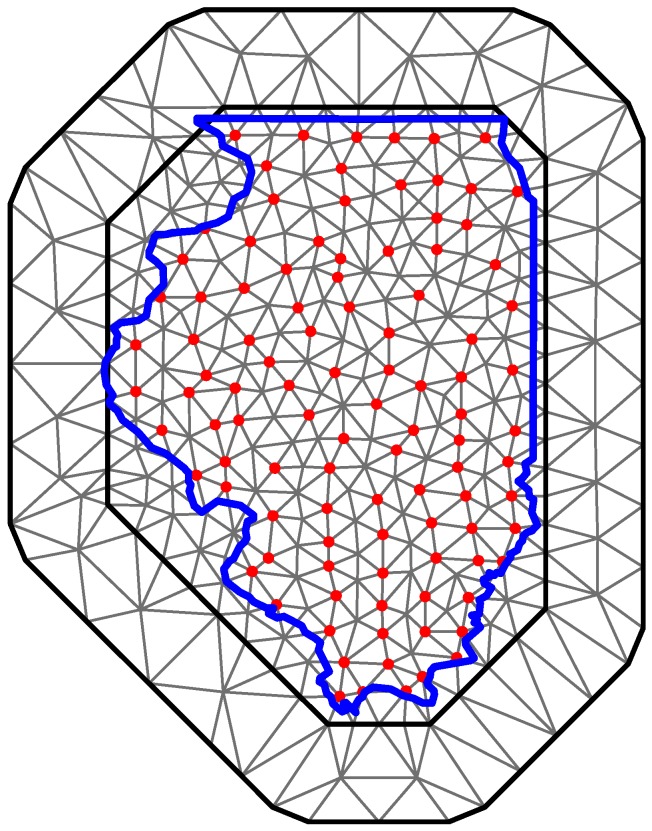
INLA mesh for the study region, blue line represents the border for the state of Illinois, and red dots represent the coordinates for the county seats.

We fit the models using R-INLA and the stochastic partial differential equations (SPDE) approach based on a Matérn covariance function [29]. As explained previously, INLA provides approximate solutions for the Bayesian problem. Figure 2 shows the mesh that is used in INLA/SPDE to approximate the spatial fields. Note that in order to increase approximation accuracy and avoid “edge effect” issues, the mesh was extended beyond the borders of the study region. R code is provided in Supplementary File 1.

## 4. Results and Discussion

We conducted model selection based on DIC values (see Table 1). The spatial zero-inflated Poisson has the lowest DIC value, however, the DIC value for the spatial hurdle model with regression for the zero-inflation probability is not substantially different from the DIC value of the zero-inflated Poisson model (the difference is less than 10%). It should be noted that the inferential results for the coefficient estimates are comparable among all three models. We also considered negative binomial models (both hurdle and zero-inflated versions), however in both cases, the performance of the model based on Poisson was superior (as measured by DIC).

**Table 1:** Model selection results based on DIC values.

Model	DIC	*Effective p*
Spatial Poisson Hurdle	404	42.94
Spatial Zero-Inflated Poisson	360	48.54
Spatial Poisson Hurdle with Probability Model	380	44.84
Spatial Negative Binomial Hurdle	459	11.85
Spatial Zero-Inflated Negative Binomial	420	11.53
Spatial Neg. Bin. Hurdle with Probability Model	435	13.84

The main difference between the Poisson hurdle model and the ZIP model is that in the hurdle model all the zero values (about 43% of the data) are assumed to be generated by the zero-generating process as oppose to a portion of the zero values in the ZIP (*i.e.*, ZIP model considers a proportion of the zero values to be generated by the Poisson probability model). As mentioned previously, the difference between the two models is subtle, and the inferential results are often very similar. In this case, we consider the hurdle model to be more relevant since it is more in line with our assumptions about the data. Thus, for the remainder of the paper, we will discuss the results from the spatial Poisson hurdle model with regression model for the zero-inflation probability.

Table 2 shows the results for the regression coefficients. Elevation is significant in both parts of the model (log-linear model for the intensity of the truncated Poisson and logistic regression for the zero-inflation probability). Population per square mile is not significant in either model. Elevation has a positive effect for the non-zero counts (*i.e.*, higher counts of Lyme disease are associated with higher elevation) and it has a negative effect for the zero-inflation probability (*i.e.*, higher probability of observing a zero count is associated with lower elevation). These results are in line with previous studies regarding the positive association of elevation with prevalence of Lyme disease (e.g., see [30]). This result is especially intuitive for our problem since most of the high-elevation areas of Illinois are in the Northern part of the state where Lyme disease is also most common. Although, population per square mile is not statistically significant, we include it in the model to adjust for differences in population distribution (which has a wide range of variability in population) and geographical size of the counties. Of course, other factors especially environmental factors should be considered for a thorough modeling of these data. Here, we limit our analysis to a simple model. Finally, note that we assumed the unreported cases in the CDC data to be zeros. This seems to be a reasonable and standard assumption, however, the validity of our analysis will be undermined, if this assumption is not true.

**Table 2:** Model results for the spatial Poisson hurdle model (with regression for probability).

Coefficient	Mean	Standard Deviation	95% *CI*
*Truncated Poisson*			
Intercept	−3.2931	1.6830	(−6.6478, −0.0008)
Elevation	0.0051	0.0019	(0.0014, 0.0089)
Population per square mile	−0.0007	0.0056	(−0.0120, 0.0102)
*Zero-Inflation Probability*			
Intercept	7.4643	1.8093	(4.1338, 11.2494)
Elevation	−0.0097	0.0022	(−0.0143, −0.0056)
Population per square mile	−0.0025	0.0086	(−0.0196, 0.0143)

**Figure 3:**
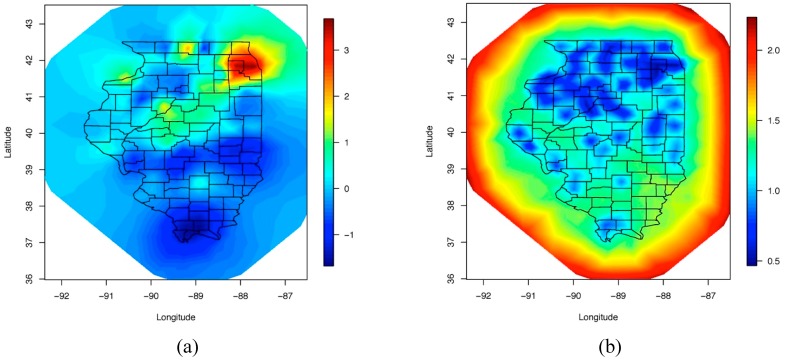
Spatial fields (**a**) posterior mean, (**b**) posterior standard deviation.

Figure 3 shows the posterior mean and posterior standard deviations for the spatial field. The smooth mean spatial field identifies the high activity areas of Lyme disease in Illinois, mostly in the Northern parts of the state (along with the standard deviation of the spatial field which shows higher uncertainty in the southern parts of the state). Note that, as previously mentioned, in order to avoid “edge effect” issues, the INLA/SPDE mesh was extended beyond the spatial domain for which we have data (See Figure 2). The effect of this choice is apparent in our results (see Figure 3a,b). In particular, in Figure 3a the posterior mean for the spatial field is extrapolated beyond Illinois borderlines. The extrapolated results are unrealistic and not based on observed data, and thus, should be ignored. This is also portrayed by the relatively high levels of posterior standard deviation at the boundaries for the mesh (see Figure 3b).

Finally, it should be noted that although both the hurdle and zero-inflated modeling frameworks are very popular and have proved to be useful in many application areas, there are several limitations and disadvantages of these methods. In particular, for both models, parameter estimation and interpretation of parameter estimates is less than ideal due to their “mixture” nature. For example, compared to alternative methods based on “regular” distributions which may handle over-dispersion caused by excess zeros (e.g., negative binomial), there are more parameters to estimate for hurdle/zero-inflated models and it is sometimes difficult to interpret potential differences in the estimated parameters for the same variables in zero and non-zero components of these models. This is not to say that distributions such as the negative binomial generally provide better alternative approaches. Rather, one should carefully explore all the “regular” and hurdle/zero-inflated approaches before finalizing the “best” choice. Also, both models are based on assumptions regarding the zero generating mechanisms and these assumptions are impossible (or at best very difficult) to validate.

## 5. Conclusions

Spatial and spatio-temporal hurdle and zero-inflated models provide flexible and useful frameworks for modeling epidemiological data with excess zeros. We anticipate these modeling options to become increasingly popular due to recent development of straightforward implementations using popular software packages, as well as increase in availability of geographically referenced data. These tools allow the researchers to avoid making limiting assumptions about the data and conduct statistical modeling based on methods which are more in line with the nature of the data.

## Supplementary Files

Supplementary File 1
