# Supplementary material for: Spatial and Spatio-Temporal Models for Modeling Epidemiological Data with Excess Zeros
Source: Int J Environ Res Public Health. 2015 Aug 28;12(9):10536–48. doi: 10.3390/ijerph120910536 (PMC4586626; doi:10.3390/ijerph120910536)
Supplement: Supplementary File 1 [file ijerph-12-10536-s001.pdf]

Supplementary Information

Spatial and Spatio-Temporal Models for Modeling Epidemiological Data with Excess Zeros

---

Ali Arab

```
## Define Mesh using Coordinates
mesh <- inla.mesh.2d(coords, max.edge=c(0.45,1))
spde = inla.spde2.matern(mesh)
data$hhid = mesh$idx$loc
## Spatial Poisson Hurdle (without model on probability)
fit <- inla(count07.11~ elevation + pop.per.sqm + f(data$hhid, model = spde) + f(u,model = “iid”),
data = data, family = “zeroinflated.poisson0”, control.compute = list(dic = TRUE,cpo = TRUE))
## Spatial Zero-Inflated Poisson
fit.zip <- inla(count~ elevation + pop.per.sqm + f(u, model = spde) + f(u,model = “iid”), data = data,
family = “zeroinflated.poisson1”, control.inla = list(h = 0.1), control.compute = list(dic = TRUE,
cpo = TRUE))
## Spatial Poisson Hurdle with Logistic Regression ## for the Zero Probability
count.ind<-rep(0,n);
count.ind[which(count == 0)]<-1
n<-length(count)
n.z<-n
ftmp<-which(count > 0)
n.y<-length(ftmp)
ytmp =rep(NA, 2 × (n + n.y))
Y = matrix(ytmp, ncol = 2)
Y[1:n, 1] = count.ind
Y[(n + 1):(n + n.y),2 ] = count[ftmp]
mu.z = rep(1:0, c(n, n.y))
mu.y = rep(0:1, c(n, n.y))
elev.z<- c(elevation, rep(NA,n.y))
elev.y<-c(rep(NA, n), elevation[ftmp])
popsqm.z<- c(pop.per.sqm, rep(NA,n.y))
popsqm.y<- c(rep(NA, n), pop.per.sqm[ftmp])
U = c(rep(NA, n.z), rep(1:n.y))
S = c(rep(NA, n.z),data$hhid[ftmp])
ldat = list( Y = Y,  mu.z = rep(1:0, c(n, n)),  mu.y = rep(0:1, c(n, n)), f.u = f(U,model = “iid” ),
f.s = f(S, model = spde), elev.y = elev.y, elev.z = elev.z, popsqm.z = popsqm.z, popsqm.y = popsqm.y)
fit<- inla(Y ~ 0 + mu.z + elev.z+popsqm.z + mu.y + elev.y + popsqm.y + f(U,model = “iid”) + f(S,
model = spde), data = ldat, family = c(“binomial”, “zeroinflated.poisson0”), control.family = list(
list(), list(hyper = list( prob = list( initial = −20, fixed = TRUE)))) ,
control.compute=list(dic=TRUE,cpo=TRUE) )
round (fit$summary.fix, 4)
fit$dic$dic
fit$dic$p.eff
fit$dic$family.dic
fit$dic$family.p.eff
# The spatial hierarchical hurdle/zero-inflated models for the Illinois Lyme Disease data
```

Stage 1. Data Model

$$Y_i \sim ZIP(y_i \mid \lambda_i, p_i) \quad \text{or} \quad Y_i \sim \begin{cases} Poisson(\lambda_i) & \text{w.p.} \quad p_i \\ 0 & \text{w.p.} \quad 1-p_i \end{cases}, \quad i = 1, \ldots, n,$$

or

$$Y_i \sim PoiHurdle(y_i \mid \lambda_i, p_i) \quad \text{or} \quad Y_i \sim \begin{cases} TruncatedPoisson(\lambda_i) & \text{w.p.} \quad 1-p_i \\ 0 & \text{w.p.} \quad p_i \end{cases}, \quad i = 1, \ldots, n,$$

Stage 2. Process Model

$$\log(\lambda_i) = \beta_0 + \beta_1 X_{i,Elevation} + \beta_2 X_{i,pop.sq.m} + \gamma_i,$$
$$\text{logit}(p_i) = \alpha_0 + \alpha_1 X_{1i} + \alpha_2 X_{i,pop.sq.m},$$

Stage 3. Parameter Models

Define prior distributions for unknown parameters:

$$\beta_i \sim N(0, \sigma_\beta^2)$$
$$\alpha_i \sim N(0, \sigma_\alpha^2)$$

Define model for spatial random effects:

$$\gamma \sim N(0, \Sigma),$$
$$\Sigma = f(\sigma^2, \tau),$$

where $f(.)$ is a spatial covariance function (e.g., exponential or Matérn).

Also assign prior densities to unknown parameters (e.g., flat gamma densities τ, and σ^2).

Note that we use "INLA" package in the statistical software R and thus, the code does not show the stages of the hierarchical model in details.

© 2015 by the authors; licensee MDPI, Basel, Switzerland. This article is an open access article distributed under the terms and conditions of the Creative Commons Attribution license (http://creativecommons.org/licenses/by/4.0/).
